# Digitally enabled decentralised research: opportunities to improve the efficiency of clinical trials and observational studies

**DOI:** 10.1136/bmjebm-2023-112253

**Published:** 2023-02-21

**Authors:** Olalekan Lee Aiyegbusi, Elin Haf Davies, Puja Myles, Tim Williams, Chris Frost, Shamil Haroon, Sarah E Hughes, Roger Wilson, Christel McMullan, Anuradhaa Subramanian, Krishnarajah Nirantharakumar, Melanie J Calvert

**Affiliations:** 1 Institute of Applied Health Research, University of Birmingham, Birmingham, UK; 2 Centre for Patient Reported Outcome Research, University of Birmingham College of Medical and Dental Sciences, Birmingham, UK; 3 Aparito, Wrexham, UK; 4 Clinical Practice Research Datalink, Medicines and Healthcare Products Regulatory Agency, London, UK

**Keywords:** COVID-19, Information technology, Drug Discovery, Drug Development

## Background

Clinical research is often site-centric, adhering to schedules largely driven by tradition and operational convenience rather than the natural history of diseases, and the diversity of target populations.^1^ It stands to reason therefore that the patients recruited to clinical trials do not always reflect real-world clinical presentations, which in turn can bias the findings and limit their applicability to real-world settings. However, in reality, few studies are totally site-centric with treatment often self-administered at home, between study-site visits; some data capture may also occur between site visits—for example, patient-reported outcomes (PROs).

Restrictions on movement imposed during the COVID-19 pandemic, disrupted many site-centric clinical trials and accelerated the interest in and growth of digitally enabled clinical research including trials.^2 3^ Challenges previously perceived as insurmountable were overcome in weeks as ethics committees, regulators, study sponsors, clinicians and patients alike, embraced new approaches like digitally enabled screening, recruitment, remote consent and data capture that were able to provide assurances that rigour and patient safety would not be compromised.

The US Food and Drugs Administration (FDA) defines decentralised clinical trials as trials executed through telemedicine and mobile or local healthcare providers, using procedures that vary from the traditional clinical trial model.^4^ Patient recruitment, delivery and administration of study medication, and collection of study outcomes data occur without in-person contact between the study team and patients in fully decentralised trials.^2^ This definition could be expanded to encompass non-trial clinical research. In this article, we explain these innovations and limitations.

## Pros and cons

### Patient recruitment and consent

Traditional site-centric clinical research recruitment involves screening and recruitment of patients at designated study centres which are typically located at sites easily accessible by clinical research staff, for example, within or close to primary or secondary care settings. This may enable more detailed screening assessments, especially involving clinical measurements at the point of recruitment, but limits participation to patients who reside or work in the vicinity, or those who are able to travel long distances to the recruitment centre. Consent is obtained in-person by study staff, ensuring that potential participants fully understand the study aims, procedures and implications of participation. Patients can have a dialogue with the study staff and ask for clarifications.

Digitally enabled recruitment includes recruitment via social media platforms or websites. This can potentially be improved by using routinely collected patient healthcare records to find eligible study participants meeting prespecified selection criteria using centralised searches. This list of prescreened patients can then be further screened either prior to or after the invitation to the study. In the model of National Health Service (NHS) DigiTrials Health Data Research Hub for Clinical Trials, invitations are sent to potentially eligible patients who then report for further screening to recruitment centres.^4^ Another model is the Clinical Practice Research Datalink (CPRD), in which a pseudonymised primary care patient database is centrally searched for eligible patients so that general practitioners in CPRD’s network can be provided a list of prescreened patients for clinical review prior to sending out invitations.^5^ Searches for patients can be refined to focus on defined geographical regions and areas of socioeconomic deprivation to facilitate wider participation of individuals from ‘underserved populations’. This could include, for example, people living with rare diseases, with complex life-limiting disabilities or in remote locations, for whom accessing a research centre may be particularly difficult and concerning. However, key considerations are needed to avoid health inequities (see table 1). Site engagement activities such as webinars are necessary to optimise recruitment.

**Table 1:** Key considerations to facilitate the participation of underserved populations in digitally enabled clinical research

Underserved groups	Considerations
Age extremes (eg, under 18 and over 75)	Consider age-related needs including access to and technological preferences Requirements for age-appropriate validated outcome assessments Consider alternative modes of delivery—such as ‘bring your own device’ Offer web-based or telephone completion for those without smartphones Consider issues of dexterity Provide training and support
Ethnic minority groups	Use culturally validated assessment tools Consider cultural requirements in development of the research and digitised system
Socioeconomically disadvantaged/unemployed/low income	Consider alternative modes of delivery—bring your own device Offer web-based or telephone completion for those without smartphones
Language barriers	Use validated translations Requirement for translator (including sign language) for interviewer-led completion
Educational disadvantage	Ensure content and training is easy to understand by participants with different educational experience
People living in remote areas	Internet access may prohibit digitised approaches. Consider backup approaches to assessment such as interviewer completion by landline
Disabilities	Consider proxy completion for those with cognitive impairment Use accessible formats to facilitate use by individuals with motor impairments

Digitally enabled approaches need to actively consider ways to make research more accessible and ensure that study cohorts are more representative of the target population, study findings are enriched, and better-quality data are available to inform the care of diverse patients. Many barriers to participation, including lack of sufficient knowledge about trial participation and concerns about treatment toxicity, would not be mitigated by a remotely delivered trial. Furthermore, protocols need to be in place to prevent ineligible or potentially fraudulent participants from signing up for studies, especially when financial incentives are in place.

Patients could still be required to visit a study site in person for final review, consent and if needed, baseline testing. However, during the COVID-19 pandemic, remote consent options like e-consent were explored. Software platforms enabling the digitisation of clinical trials, including the capture of patient generated data (eg, electronic PROs (ePROs), or eClinical Outcome Assessments now also have integrated eSignature capabilities as part of the informed consent process, the flow of which can be adapted according to study specific requirements. In a National Institute for Health and Care Research (NIHR)-funded study of non-pharmacological interventions to manage Long Covid, study participants provided eSignature with double verification (email) to take part in the study.^6^

### Data collection

Data collection for site-centric study approaches can guarantee standardised approaches to measurement. Furthermore, certain assessments may be difficult to replace with self-assessments at home and undermine the rigour and reproducibility of results.^3^ However, site-centric approaches may be more costly in terms of resources, such as staff and patient time and carbon footprint, and a potentially narrower participant pool.

Digitally enabled studies may include outcome assessment and passive long-term follow-up using routinely collected electronic healthcare record (EHR) data. This not only reduces costs associated with data collection, but also minimises lost to follow-up assuming participants have not withdrawn from the study. Health data linkage is a method of gathering information from distinct sources (eg, hospital registries, administrative claims) about the same patient or group to create a more comprehensive set of data.^7^ The disadvantage is that not all outcomes can be suitably assessed using EHR data and an a priori decision would be needed on how any given outcome is being defined by generating lists of all possible codes related to it.^8 9^ Furthermore, they may not be standardised as the data is not primarily collected for research purposes.

Digital Health Technologies (DHTs) capturing digital biomarkers ‘objective measures of physiological, pathological, anatomic, behavioural, social or activity characteristics, and patient self-report using digital technology’ are promising new tools for advancing precision medicine and supporting clinical trials.^1 3 10^ While many digital biomarkers are still being validated, in future, they may provide detailed information on physiological processes and explain, influence and/or predict health-related outcomes.^11 12^ They may also inform diagnostics, dosing titration and serve as endpoints for clinical trials.^1 13^

Patient-generated health data, including PROs,^14^ are an integral part of digitally enabled clinical research. Increased use of internet access, home computing and smartphones, in combination with wearables, and biosensor technology provides the opportunity to remotely collect patient-generated health data that are closer to the real-world daily contexts of patients while still allowing for rigorous control, support and management.^15^ Such technology enables participants to provide data at a time which best suits them, thus potentially maintaining participant motivation and retention.

PROs capture patient’s perspectives, contributing to a more holistic and comprehensive assessment of the safety, efficacy and tolerability of interventions under investigation.^16^ PROs have traditionally been measured using paper-based questionnaires completed by participants while at site visits. However, technological advances have facilitated the development of ePROs.^17^ Advantages of using ePROs include reduction of administrative and respondent burden, reduced data entry errors and real-time data collection, which could allow patients to remain at home. Furthermore, ePROs can be configured to trigger notifications that alert research and clinical teams in case of important clinical symptoms. These alerts can prompt more timely clinical intervention and onward referral to specialist care if required as exemplified in the Therapies for Long Covid study (b ox 1).

Box 1 **Overview of the Therapies for Long Covid (TLC) study**

Led by researchers from the University of Birmingham the TLC Study aims to identify and recruit non-hospitalised patients with Long COVID with symptoms lasting 12 weeks or more to a major digitally enabled clinical study using CPRD primary care data.^6^ The TLC study links to a digital platform (Atom5) to facilitate the capture of patient-generated data including self-report symptoms, quality of life and work capability.^6 17^ A subgroup of patients are providing blood and other biological tests, with a single site visit, to understand the immunology of Long COVID, and will wear a device to remotely measure their heart rate, oxygen saturation, step count and sleep quality.

CPRD, Clinical Practice Research Datalink.

## Regulatory considerations

Regulatory aspects of clinical studies including trials for site-centric research are well established so this section will focus on the key considerations for digitally enabled clinical research. The MHRA’s Delivery Plan 2021–2023 outlines a commitment to innovative clinical trials for the immediate future.^18^ The European Medicine Agency Network Strategy to 2025 also encourages the use of digital tools in clinical research and use of real-world data.^19^ The FDA’s Digital Health Center of Excellence has also produced a series of guidance documents on DHTs.^20^

Ensuring the integrity, reliability and robustness of data generated by digitally enabled decentralised research is essential. Clear processes need to be in place to identify and verify sites and participants. Vouchers, which may be offered for participation could incentivise not only participants but also ‘scammers’ so strategies should be introduced to mitigate such risks. Guidance from regulators in the UK and Europe has been developed.^21 22^ Codevelopment of systems with patient partners and usability testing with representative patients will help ensure that systems meet participant needs.

## Patient perspectives on site-centric versus digitally enabled clinical research

In 2021, James Lind Care undertook an online survey and interviews with 874 community members across the UK and Denmark to understand patients’ preferences, needs and expectations of participating in a decentralised clinical trial.^23^ Most of the survey respondents preferred decentralised trials while only 12% favoured the classical design.^23^ Ninety-two per cent of the respondents were willing to wear digital equipment such as sensors or smartwatches. While study participants may be highly motivated to enrol for decentralised trials, maintaining motivation and retention may be more difficult due to a reduction in direct contact with clinical staff. Patients may worry about their capability of managing wearables and devices,^23^ but this can be mitigated through service solutions.

## The right approach for each study

A digitally enabled decentralised approach offers an opportunity to open participation in clinical research to a much wider population and in settings that are more representative of the context within which interventions will ultimately be used. Ultimately, for any given clinical trial the specific characteristic of the intervention, population and the trials objectives will dictate what methods are used and decentralisation together with use of routinely collected data and PROs will be a part of the available toolset, which will shape the ongoing and future landscape of clinical and epidemiological research.
